# Compliance Testing of Hemp (*Cannabis sativa* L.) Cultivars for Total Delta-9 THC and Total CBD Using Gas Chromatography with Flame Ionization Detection

**DOI:** 10.3390/plants13040519

**Published:** 2024-02-14

**Authors:** Terri L. Arsenault, Kitty Prapayotin-Riveros, Michael A. Ammirata, Jason C. White, Christian O. Dimkpa

**Affiliations:** Department of Analytical Chemistry, The Connecticut Agricultural Experiment Station, New Haven, CT 06511, USA; kitty.prapayotin-riveros@ct.gov (K.P.-R.); michael.ammirata@ct.gov (M.A.A.); jason.white@ct.gov (J.C.W.); christian.dimkpa@ct.gov (C.O.D.)

**Keywords:** *Cannabis sativa*, cannabidiol, compliance testing, hemp, gas chromatography, regulatory testing, tetrahydrocannabinol

## Abstract

The United States Agriculture Improvement Act passed in December of 2018 legalized the growing of *Cannabis sativa* containing not more than 0.3% total Delta-9 tetrahydrocannabinol (THC) in the country. While *Cannabis sativa* has been cultivated for hundreds of years, the illegal status of the plant in the United States, and elsewhere, has hindered the development of plant cultivars that meet this legal definition. To assess sampling strategies, and conformance to the THC limit, 14 cultivars of hemp were grown and tested by using gas chromatography with flame ionization detection for total delta-9 THC and total cannabidiol (CBD) during 2020, 2021 and 2022. Each year, samples of fresh plant material were collected from each cultivar weekly, beginning in mid-August and ending in late October, to examine the rate of increase in THC and CBD for different cultivars and select individual plants. The sampling demonstrated that both CBD and THC increase rapidly over a 1–2-week time frame with maximum concentrations (about 16% and 0.6%, respectively) around late September to early October. The testing of individual plants on the same day for select cultivars showed that while the ratio of CBD to THC remains constant (about 20:1 in compliant hemp) during the growing season, the individual plants are highly variable in concentration. Whereas previous studies have shown cultivar-dependent variability in THC production, this study demonstrated a novel plant-to-plant variability in the levels of THC within the same hemp cultivar. Understanding variability within and between hemp cultivars is useful to determine field sampling strategies and to assess the risk of crop embargoes to growers by compliance regulators.

## 1. Introduction

*Cannabis sativa* has been cultivated around the world for centuries [1,2]. Originally, cultivation may have been predominantly for fiber, but historical records indicate contemporaneous medicinal use for ailments like rheumatic pain, constipation and malaria [3,4,5,6]. Evidence suggests that psychotropic use of Cannabis began in the early 1600s in North America with medicinal use following in the 1800s for ailments such as tetanus, epilepsy and rabies and as a muscle relaxant [7]. In the United States, the Controlled Substance Act of 1970 created five schedules of controlled substances primarily based on accepted medical use and the potential for abuse [8]. Schedule I drugs are defined as having no currently accepted medical use and a high potential for abuse. Schedule II drugs have accepted medical use, but with a high potential for abuse, and Schedule V drugs have accepted medical use and a much lower potential for abuse. When the US law was enacted, there was no accepted medical use and fears about abuse, so both marijuana (*Cannabis*, marihuana) and the tetrahydrocannabinols (THC, delta-8 THC, delta-9 THC, dronabinol and others) were put on Schedule I, which controls the possession of both the plant and the chemical. Recent documentation of numerous medical uses such as for nausea/vomiting, pain, muscle spasticity, mood disorders, etc., combined with limited potential for abuse, suggest similarity to Schedule III drugs [9].

The Agriculture Improvement Act of 2018, otherwise known as the Farm Bill [10], legalized the nationwide cultivation of *Cannabis sativa* containing ≤0.3% total delta-9 THC on a dry weight basis, designated as hemp, while maintaining *Cannabis sativa* > 0.3% total delta-9 THC, designated as marijuana, as still illegal in the United States, as it is in many countries globally [11,12]. Following legalization in the US and other countries, it has been estimated that around 147 million people consume the plant globally, mainly for its THC-derived effect [13]. In the US Farm Bill, hemp growers are required to have plant material collected up to 30 days before harvest, and test results must show compliance with the 0.3% regulatory threshold. Crops with THC levels exceeding the threshold are to be destroyed. Notably, the law specifically states that the method of analysis for compliance testing must include the postdecarboxylation products. This stipulation requires the primary plant product of tetrahydrocannabinolic acid (THC-A) to be added to the Delta-9 tetrahydrocannabinol for a total Delta-9 THC concentration. Gas chromatography utilizes hot injection (250 °C), which decarboxylates THC-A in situ and produces one test result. Liquid chromatography utilizes a lower temperature injection, producing a result for THC and THC-A separately, which are combined by formula to produce one test result designated as total THC. The ratio of THC to THC-A is irrelevant for compliance purposes relative to the Farm Bill. While the Farm Bill removed hemp from the Schedule I list, it did not at the time specify what may be done with the harvested commodity [10]. Those consumer products must still meet regulatory requirements issued by the United States Food and Drug Administration (FDA) and/or Drug Enforcement Administration (DEA) to be moved between states and may have additional requirements within states [10].

The biosynthesis of THC and CBD is related by shared pathways involving the activities of a cannabidiolic acid synthase, a cannabichromenic acid synthase and a tetrahydrocannabinolic acid synthase. Respectively, these enzymes convert a common precursor, cannabigerolic acid, into the acidic forms of cannabidiol, cannabichromene and tetrahydrocannabinol [14,15]. With respect to these cannabinoids, the difference between hemp and marijuana is the genetic coding for enzymes that produce either THC-A or cannabidiolic acid (CBD-A) from the precursor molecule cannabigerolic acid, and the only reliable method to distinguish the two analytes is with laboratory testing. *Cannabis sativa* cultivars are sometimes described in terms of chemotypes: chemotype I is predominantly THC (marijuana), chemotype II is predominantly CBD with moderate THC and chemotype III is predominantly CBD with low THC (hemp) [16,17]. The previous illegal status of the plant has meant that the limited knowledge about breeding, varietal differences, growth conditions, etc., has restricted many types of scientific study until recently [18,19]. Furthermore, though it remains federally illegal in the United States, marijuana has been legalized in many states for medicinal and/or adult use. In 2012, Colorado and Washington became the first states to legalize Cannabis. By 2023, 38 states had some form of legalized medical cannabis, which included 24 states with fully legalized adult use [20]. With increases in indoor and outdoor cultivation, and as a wind-pollinated crop, the development of hemp seed may be complicated by both feral and cultivated marijuana.

As required by the Farm Bill, the United States Department of Agriculture (USDA) Agricultural Marketing Service (AMS) published a final rule, effective on 22 March 2021, that established a national regulatory framework for hemp production in the United States [21]. The final rule requires test samples for each field lot to be collected within 30 days of harvest for compliance testing. A lot is defined as a contiguous growing area containing the same cultivar of cannabis throughout and could be a field or greenhouse location. Sampling agents are directed to cut 5–8 inches from the terminus of branches to represent a lot, and the USDA standard sampling protocol states “The standard sampling protocol ensures, at a confidence of 95 percent, that no more than one percent of the plants in each lot would exceed the acceptable hemp THC level and ensures that a collected sample represents a homogeneous composition of the lot” [22]. The document contains formulas to calculate the number of primary plants to sample for field lots larger than ten acres, and for lots smaller than ten acres, an “example 2” is provided that specifies a minimum sample size of one plant per acre. In the US, the State of Connecticut (CT) had 109 licensed producers of hemp in 2019, which increased to 140 in 2020 but then decreased to 98 in 2021 and 78 in 2022. The average lot size has ranged from 1.44 to 0.56 acres, so for most lots in CT, the final rule allows a lot to be represented by only one or two plants [23].

As an ISO/IEC 17025:2017 state testing laboratory, the Analytical Chemistry laboratory of the Connecticut Agricultural Experiment Station (CAES) is suited to address how field sampling affects both THC and CBD test results. The laboratory has access to both an experimental farm where hemp may be grown and collected and regulatory testing facilities. Testing laboratories routinely comminute and homogenize samples and can measure the uncertainty related to those processes. Sample agents develop protocols to properly collect samples in the field or greenhouse that are representative of a lot. A laboratory is usually unable to assess how test items relate back to the decision unit or the lot that the sample is meant to represent. Moreover, while the number of plants that compose a sample is estimated based on the variability between the plants, the actual variability between plants is usually unknown, making the development of the sampling protocol difficult. Therefore, while test portions should be reflective of the item delivered to the laboratory, the effect of the field collection procedure on the final test result, a potentially significant parameter, is rarely studied. Furthermore, while the AMS guidance allows a minimum sample size of one plant per lot of one acre, it provides little guidance on how to ensure that no more than one percent of the plants would exceed the acceptable hemp THC level, or how many plants are required to represent a homogeneous composition of the lot [10].

Previous studies have investigated the levels of THC and CBD in hemp cultivars over time. However, plant-to-plant differences within the cultivar have not been assessed [24,25,26]. Overall, the current study aimed at providing evidence for the occurrence of plant-to-plant differences in THC and CBD production as a function of time to support regulatory efforts. Understanding plant-to-plant variations in THC production within the same cultivar will help in the selection of hemp varieties by growers to ensure that varieties of preference are suited for maintaining the regulatory limit. Moreover, it could provide useful feedback to hemp breeders regarding the fidelity of their germplasms. To this end, gas chromatography with flame ionization detection was used to evaluate hemp plant material for their THC and CBD contents with the specific objective of examining the rate of increase in THC and CBD in different cultivars during the growing season and assessing plant-to-plant variability within the same cultivar. It is anticipated that this study will provide information that is useful to regulatory bodies ensuring compliance with federal regulations. While CBD has no bearing on the legal status of the crop in the US, understanding the relative rate of increase for THC and CBD is critical to yield calculations of compliant hemp, the creation of sampling strategies that meet the AMS guidance and ultimately the economic viability of hemp as a crop.

## 2. Results

### 2.1. Effect of Time on THC and CBD Production

**Figure 1:**
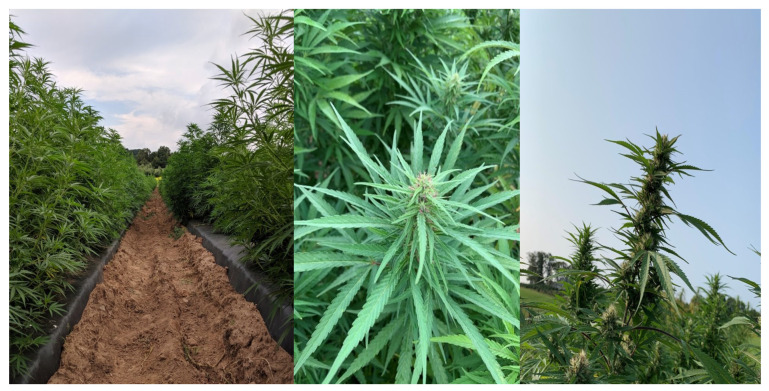
*Cannabis sativa* (hemp) plants from this study in the field plot. The left panel shows plants in and between rows; middle panel is a young bud and right panel is a mature bud.

**Figure 2:**
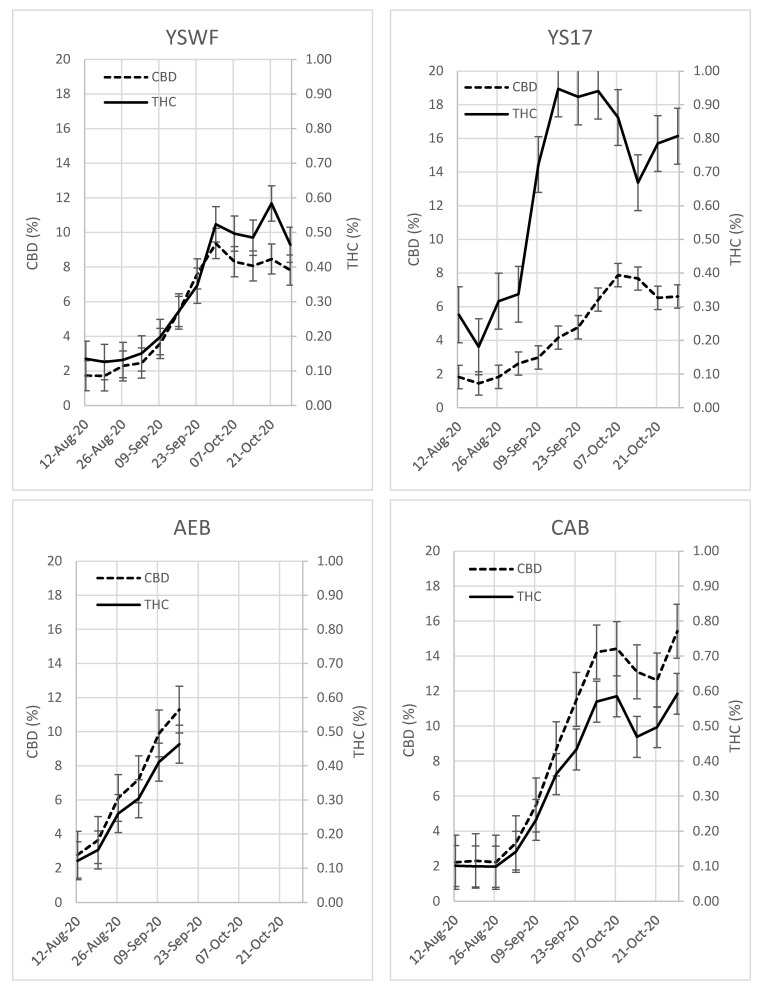

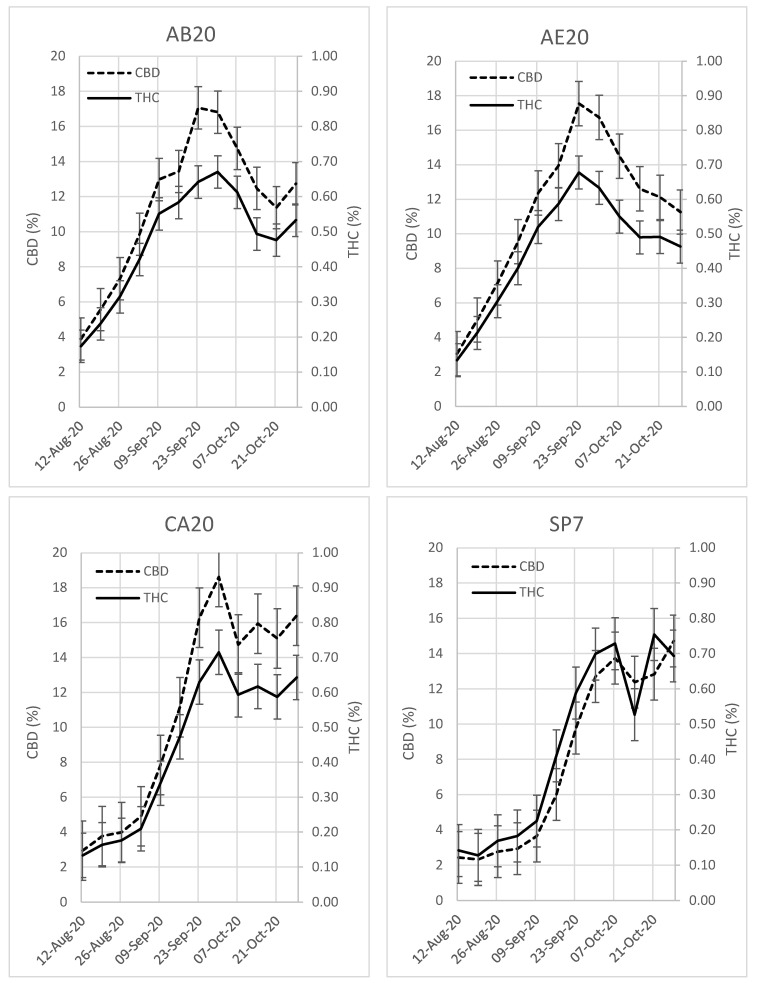
Concentrations of CBD and THC sampled weekly during the 2020 growing season. THC is displayed as a solid line with the concentration on the right axis and CBD is the dashed line with the concentration on the left axis. THC and CBD are closely correlated and increase rapidly as the flowers mature.

**Figure 3:**
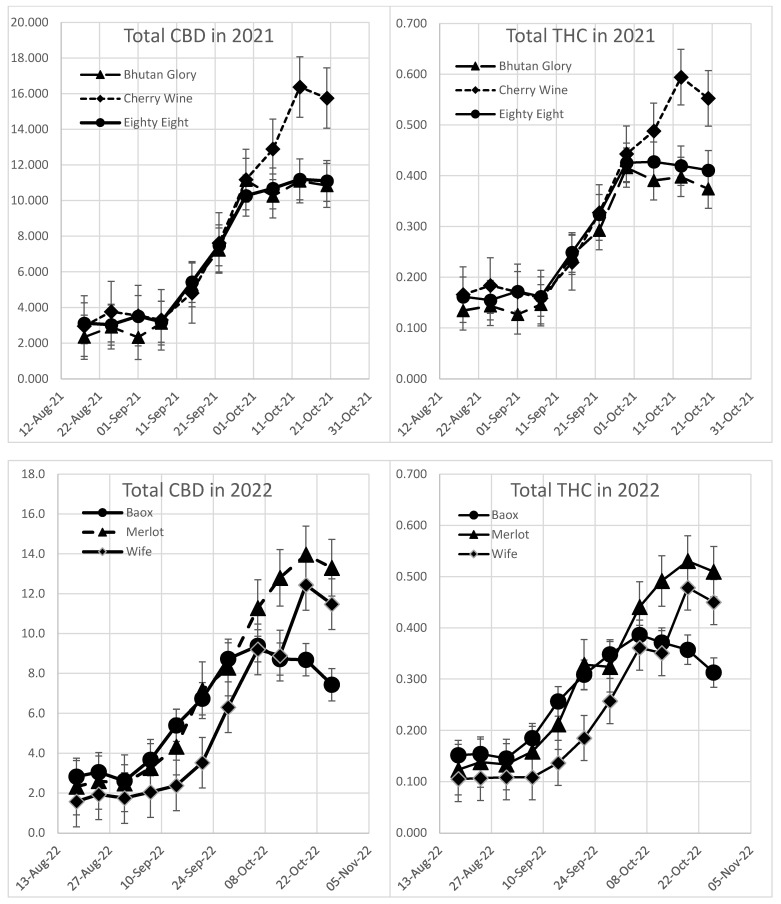
Concentration of THC and CBD during the 2021 and 2022 seasons. THC and CBD concentrations are closely correlated and began to rapidly increase in the beginning of September reaching maximal concentration in mid-October.

During the growing seasons of 2020, 2021 and 2022, fourteen cultivars of hemp were planted in the field (Figure 1). Each plant within a cultivar was sampled weekly, composited to one sample and tested for THC and CBD by using gas chromatography with flame ionization detection (Figure 2 and Figure 3). All cultivars start low for both THC and CBD in late August, begin to increase around mid-to-late September and then trend toward leveling off or decreasing through mid-October. Sampling stopped on the AEB cultivar in mid-September of 2020 because of heavy damage during a windstorm during the first week of August that left most plants withered and dead by 16 September. For 13 of 14 cultivars, the CBD and THC track similarly, with the YS17 being the exception. For all the tested cultivars, there is a steep rise in both THC and CBD content as plants mature. Every cultivar was below or near the THC compliance limit of 0.3% in early September, but all cultivars exceeded the limit within one to two weeks. USDA guidance allows laboratories to calculate and apply a measurement uncertainty that is applied to the test result, and crops are embargoed (or remediated) if the lower range is above 0.3%. This means that even if a test result is above 0.3%, they may still “pass” and growers are allowed to harvest. There is potentially a large amount of variability in the parameters and methods used by individual laboratories to calculate measurement uncertainty. There was a noticeable decrease in THC and CBD for many cultivars on 14 October 2020, which may be related to weather. There was heavy rain on both 13 and 14 October, which may have lowered the THC and CBD content, while the temperature remained relatively the same, with a high of 12 °C and a low of 5 °C. In late October, most plants were in serious decline, and sampling ceased.

**Figure 4:**
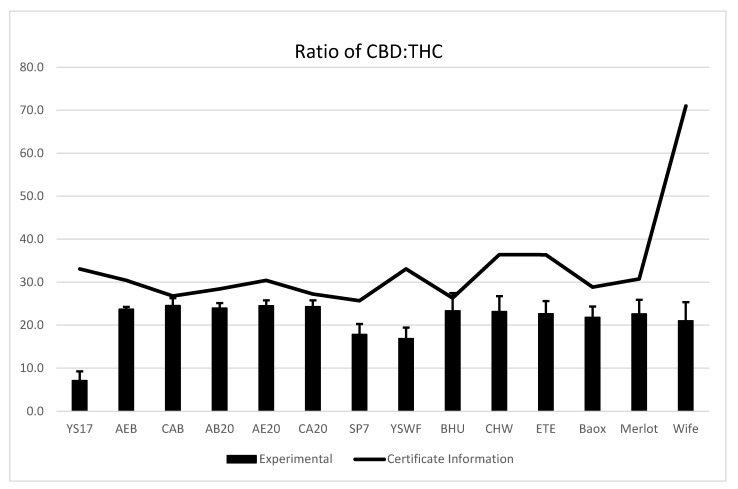
Average ratio of CBD to THC for weekly samples compared to the ratio stated on the certificate of analysis. The error bars represent standard deviation of the average for the weekly samples. In 2020, samples were collected for twelve weeks (except AEB, which was collected for 7 weeks), ten weeks in 2021 and eleven weeks in 2022. The advertised ratio of CBD to THC from the certificate was higher than the experimental ratio for all tested cultivars.

Figure 4 shows the average ratio of CBD to THC for each cultivar across the growing seasons compared to the ratio listed based on the certificate of analysis provided with the seeds. The error bars on the columns represent the standard deviation for the average across the growing season. Notably, the Wife cultivar certificate of analysis listed the total CBD as 10.65% while the total delta-9 THC was listed as 0.15%, resulting in an expected ratio of 71, far higher than any other seed. In general, while the ratio is consistent across the growing season, the certificate of analysis for all cultivars overestimated the ratio of CBD to THC.

### 2.2. Effect of Plant Maturity on THC and CBD Levels

**Figure 5:**
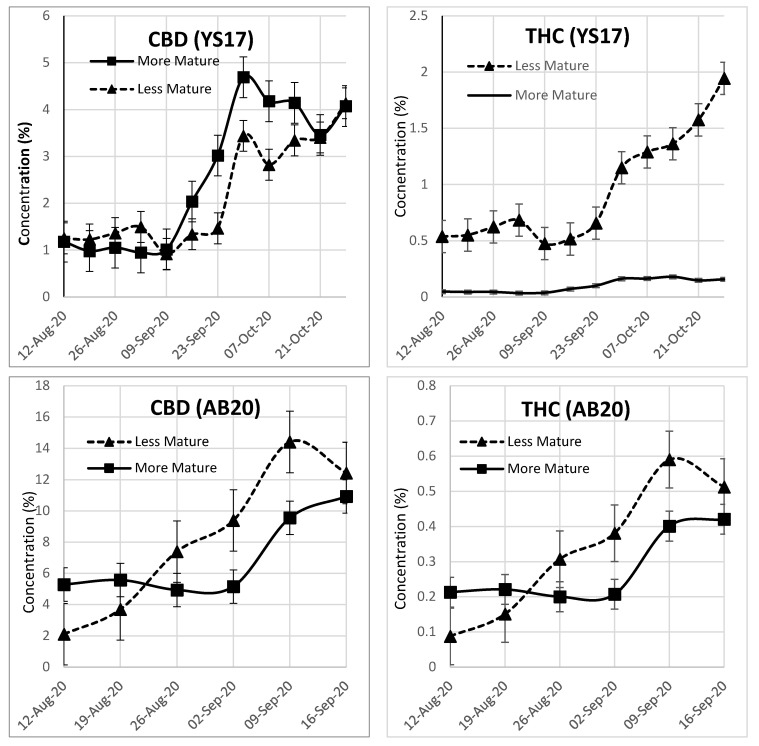
CBD and THC concentration in individual plants of differing initial maturity for two cultivars of hemp. The YS17 plant that was initially less mature was a chemotype II plant that far exceeded the legal limit for THC at full maturity, but the steep rise in both THC and CBD content is evident. The AB20 plant showed a similar steep rise, at virtually the same rate, for both THC and CBD.

The comparison of individual plants from the YS17 and AB20 cultivars of different levels of maturity when initially sampled is shown in Figure 5. When sampling began, the more mature plants had noticeable buds while the less mature plants had smaller or nonexistent buds. For the YS17 pair, the CBD content at the first sampling of the plant that looked noticeably more mature was similar, while the THC content of the visibly less mature plant was dramatically higher. As the season progressed, both plants reached full maturity around late September, but the plant that was visibly less mature on 12 August was almost 2% THC by 28 October, which far exceeds the legal definition of hemp. The pair of AB20 plants was tracked weekly from 12 August to 16 September, at which time wind damage ended sampling. For the first two weeks, the CBD and THC content was higher in the mature plant, but during week three, the immature plant began to increase in CBD and THC content and eventually surpassed the plant that was initially more mature. While the individual plants of the same cultivar varied in the timing of maturity, the same pattern of rapid maturation is evident.

### 2.3. Effect of Bud Location on THC and CBD Levels

**Figure 6:**
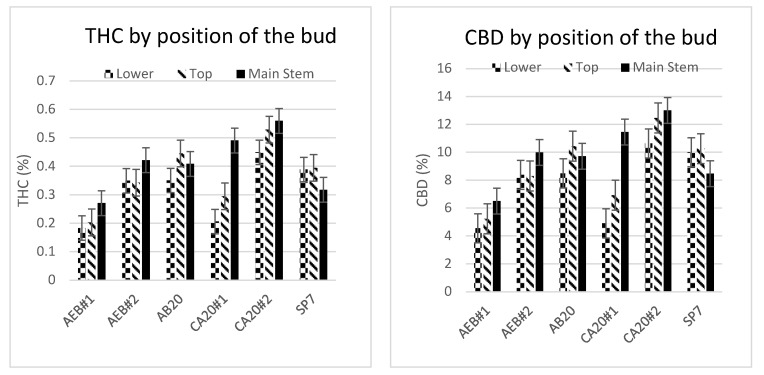
THC and CBD in buds taken from six different plants from differing positions on the plant. While no difference was found between buds taken from the main stem or top and lower tiers of the plants, the test is inconclusive.

To assess the relationship between bud location on plants with THC and CBD levels, the main stem bud was compared to the terminal-side buds from the top part of the plant and the lower portion of the same plant (Figure 6) six times in 2020. The length and diameter of the main stem bud are much larger than the other terminal buds. The average mass of the main stem bud (45.2 g) was about the same as the average mass of three terminal buds, regardless of either being samples from the middle (42.2 g) or from the bottom (39.3 g) section of the plant. The statistical analysis shows no difference in the THC or CBD content between the main stem bud and those collected from the top or lower tier. However, the power of the ANOVA for both THC and CBD (0.318 and 0.314, respectively) is below the desired power of 0.800, which indicates that an undetected difference may exist, and the results are inconclusive.

### 2.4. Assessment of Between-Plant Variability in THC and CBD Levels on Select Days

**Figure 7:**
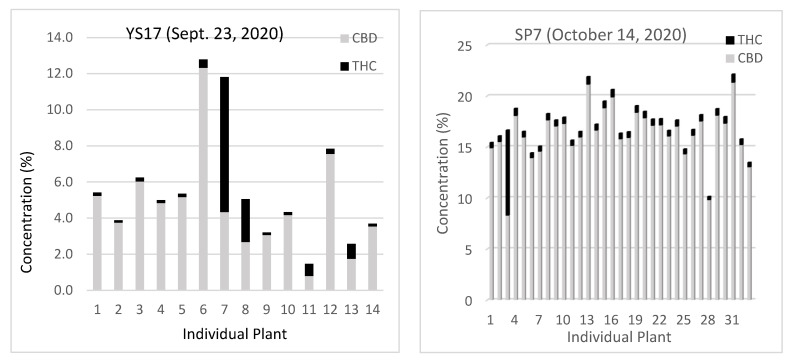

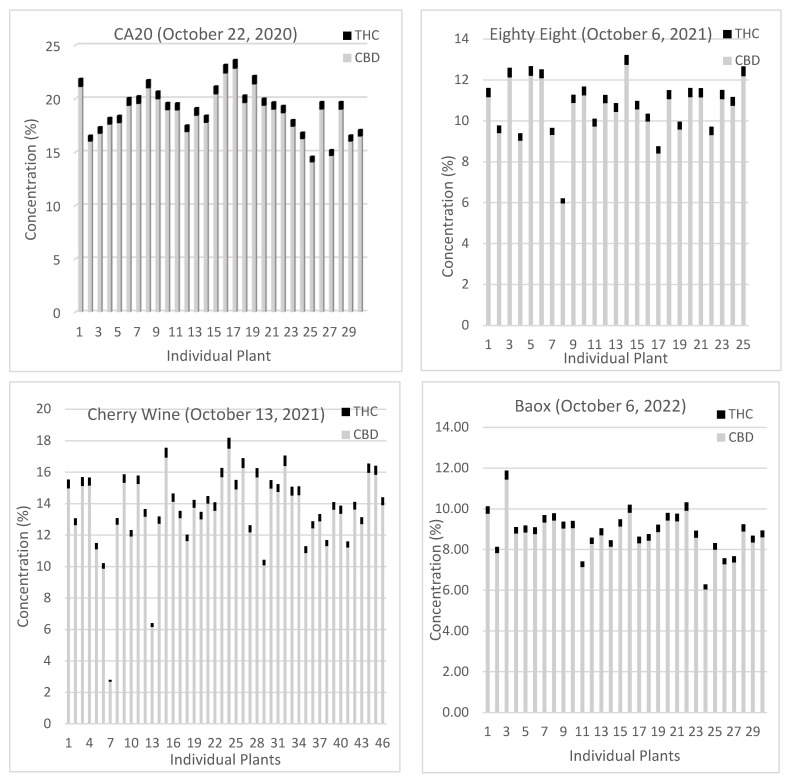
THC and CBD content of individual plants collected on the noted day.

Subsequently, every plant of the select cultivars was collected on specific days and analyzed for THC and CBD to illustrate the variability between plants on a particular day (Figure 7). The THC results are presented in Table 1 and the CBD results are presented in Table 2. Cultivar YS17 was known to be problematic based on preliminary testing conducted in 2019, and indeed further testing revealed both a high degree of variability in the total cannabinoid content and the presence of chemotype II plants. An examination of the ratio of CBD to THC shows how clearly plants 7, 8, 11 and 13 differed from the rest and were more typical of chemotype type II plants. Cultivar SP7 had one chemotype II plant, but in 2021 and 2022, all individual plants were predominantly CBD type. However, even amongst the true hemp cultivars, there was up to a 50% difference between the individual plants on a single day.

## 3. Discussion

In this work, we examined the rate of increase in THC and CBD during the hemp plant growing season and evaluated plant-to-plant variability in the levels of these metabolites to provide information with regulatory implications. Studies such as this are a natural first step to a postharvest and cannabis product development industry that complies with regulations [27]. Our data clearly demonstrate that of the fourteen tested cultivars in this study, all would exceed the THC regulatory limit of 0.3% (not considering the measurement uncertainty) when fully mature, with most by a substantial margin. This finding agrees with those of others [28,29] who reported that the total THC of several hemp cultivars exceeded the 0.3% threshold and stayed above that level for the rest of the season. Surprisingly, some hemp cultivars were found to have chemotype II plants, which are problematic and may lead to individual plants having psychoactive levels of THC. A study conducted by the University of Kentucky found that three of four tested cultivars exceeded the compliance level and that secretion color was not useful for predicting the exceedance [30]. These studies and others [25,28,31,32] showed that THC and CBD levels rise rapidly over 1–2 weeks during maturation. Notably, the Midwestern Hemp Database 2020 research report notes “The reality is that most hemp cultivars will go “hot” (>0.3% THC) if not monitored appropriately, as 25% of the samples tested were above 0.3% total THC regulatory limit” [33]. Again in 2021, the report noted that 83% of cultivars exceeded the threshold for compliant hemp by week 7. At a minimum, a certificate of analysis may not represent fully mature plants and is not sufficient for a grower to be confident that they are producing THC-compliant hemp, either as a bulk commodity or as individual plants. It is expected that hemp seed producers will produce more consistent genetics with time, but the THC is likely to continue to exceed 0.3% at full maturity.

In addition, certificates of analysis may not accurately reflect the ratio of CBD to THC. The present work and others [28,30,34] have shown that the ratio of THC to CBD is consistent during the growing season, which may be used to predict the potential yield of CBD. For example, if the ratio of CBD to THC is 25:1, then the maximum amount of CBD is 7.5% when the THC is 0.3%. Similarly, when the ratio is 30:1, the maximum CBD would be 9% when the THC is 0.3%. Early testing may be used to estimate this ratio, but the rapid increase in CBD and THC will complicate the timing of the harvest based on testing. Generally, the maximum amount of CBD for THC-compliant hemp is around 8%, which may be lower than estimated on the certificate of analysis.

The final rule in the Farm Bill allows for 30 days to occur between the collection of the test sample and the completion of the harvest; this is due to significant input that the 15 days required by the interim rule was not practical [18]. The present study suggests that THC content increases rapidly over a 1–2-week time frame, leaving a high potential for a noncompliant final product, even when the preharvest sample was compliant. A study in Virginia suggested that while CBD increased steadily over a three-week time frame, THC spiked dramatically over one week, which suggests that it is possible to time the harvest to both maximize CBD and minimize THC [35]. While the Farm Bill only requires the testing of the preharvest sample, some processors may require the testing of biomass material prior to processing, which may lead to the rejection of the postharvest material for noncompliant THC levels, even if the preharvest sample was acceptable.

Individual plants of the same cultivar mature at different rates, so the maturity of the plants constituting the preharvest test sample will influence the final test result. For instance, on 9 September, one plant of cultivar AB20 had 0.40% THC while a nearby plant had 0.59%, and one plant of cultivar YS17 had 0.48% while another had 0.04%. Therefore, one or two cuttings are probably not a homogeneous representation of one or two acres of hemp, even if they are of the same cultivar. Likewise, a different study found a significant difference in the THC content of ten individual plants sampled at the same time, even though the plants were clones and cautioned that many plant samples are necessary to accurately represent a field [36]. These field data suggest that the minimum number of plant samples should be larger than allowed by the final rule for lot sizes less than ten acres.

Ideally, sampling should reflect the decision unit. For example, if the entire plant will be shredded to make biomass, then the entire plant should constitute the analytical sample. Likewise, if hemp is sold as a smokable flower, then only the flowers would constitute the analytical sample. In practice, this becomes challenging because each lot would be sampled according to its end use, which is difficult to employ or track. Instead, the final rule specified that a sample must consist of 5–8-inch cuttings of floral material of terminal buds, main stem buds or central cola. While the current study found that the main stem bud and the terminal buds from the middle and bottom may have equivalent THC/CBD content, another study showed that sampling the top third of the plant would lead to an overestimation of THC content by nearly 37% [36]. This was potentially due to more leaf material in the longer cuttings used in that study versus the shorter cuttings used in the current work, which are almost entirely floral material. This would be especially important for labeling products sold as individual buds. More investigation is needed to define the level of variability between buds on the same plant.

The calculation of the measurement uncertainty (MU) is a routine part of the required activities for accredited laboratories, but typically the calculation applies only to the activities conducted within the laboratory. The University of Kentucky provides a hemp proficiency test (PT) for cannabinoid analysis in the fall of each year, which consists of two rounds of using two thoroughly homogenized and dried hemp plant materials; the authors participate in this PT regularly. In 2022, the test results for the four samples analyzed by 49 (first round) and 47 labs (second round) across the United States showed that the average % relative standard deviation between laboratories was 15.7% for total delta-9 THC. Using a coverage factor of two, the expanded MU is in the range of 31%, which does not include the uncertainty associated with field sampling or drying. While our laboratory measures loss on drying, it does not directly measure moisture content, and the term dry weight is not clearly defined in the USDA guidance. In addition to the field sampling strategy, preliminary data indicate that differences in drying techniques may add to the MU applied to test samples for compliance with the THC limit.

Taken together, this study demonstrated the occurrence of plant-to-plant variability within hemp cultivars that can be exceedingly high, especially in the presence of chemotype II plants predominated by CBD with moderate THC production. A previous study [30] showed cultivar differences in THC levels, and the effects of agricultural inputs such as fertilizer and pesticides on THC and CBD levels have been assessed [31,32,37], adding to the dynamics in the levels of these metabolites. However, to our knowledge, this is the first evidence of the extent of plant-to-plant variability within a hemp cultivar for THC levels. One issue with most statistical descriptors is the underlying assumption of normally distributed data, which is violated when chemotype II plants are included in the analysis. While they are outliers, they are significant for the test result and potentially of great concern for hemp producers. For instance, in cultivar SP7, the average THC was 0.86% with 157% RSD if the chemotype II plant is included (containing 8.4% THC), but the average THC was 0.63% with 15% RSD if the chemotype II plant is excluded. Excluding the two problematic cultivars, the MU associated with field sampling could be described by the %RSD times a coverage factor of two, which would be between 22% and 44% for THC depending on the cultivar. The field sampling must be a homogeneous representation of the field lot as per USDA guidance, but the selection of the most mature buds during preharvest sampling based on visual examination would be a better representation of the maximum THC/CBD potential.

## 4. Materials and Methods

### 4.1. Plant Material and Growth Conditions

Hemp seedlings were planted at the CAES experimental farm in Hamden, Connecticut, in mid-June in 2020, 2021 and 2022 (Table 3). The farm soil type for the plot is described as Cheshire fine sandy loam [38,39], and sloping at 3 to 8 percent, as per the Natural Resources Conservation Service soil survey. Table 3 lists the cultivars, all of which were claimed to be hemp or low THC cultivars, and many had a certificate of analysis stating that the THC content was below the legal limit. The Youngsim 10 was the only cultivar that was not feminized, and the male plants were culled prior to full maturity. The seeds were planted in a greenhouse and transplanted outside in about mid-June in rows spaced five feet apart with plants spaced four or five feet apart. Plastic sheeting was used to control the weeds, and drip irrigation was used as needed. All the cultivars planted were represented by between 8 and 46 individual female plants.

### 4.2. Plant Sampling for THC and CBD Determination

Unless noted differently below, sample collection for all testing consisted of using the top 2–3 inches of the terminal buds from the top tier of each plant, and they were generally collected between 9 am and 10 am. To test THC and CBD within a cultivar, a single bud from each plant of the same cultivar was compiled to generate one weekly sample from late August to late October. In 2020, to test the timing and level of CBD and THC for individual plants of the YS17 and AB20 cultivars, a plant that initially looked more mature was sampled weekly along with a nearby plant that initially looked less mature (two pairs). Also in 2020, on six occasions, a cutting of the main stem bud (5–8 inches) was compared to three cuttings from the terminal buds (2–3 inches) in the top tier of a single plant along with three cuttings from the lower tier of the same plant. Lastly, every plant within six cultivars on select days was tested separately with each plant represented by three buds.

### 4.3. Materials and Reagents

Every daily batch of samples included a laboratory blank of parsley (purchased at a local grocery store), a control sample with a well-characterized amount of THC and CBD created in house and a certified reference material of hemp (Absolute Standards, Hamden CT, USA [part No. 54999]). Samples and standards were prepared in ACS-grade methanol from Fisher Scientific, Hampton, NH, USA [part no. A452]. A mixed calibration standard containing THC, CBD and Cannabinol (CBN) was purchased from Restek, Centre County, PA, USA [part #34014], and an independent calibration standard containing only THC was purchased from Sigma-Aldrich, St. Louis, MO, USA [part no. T4764].

### 4.4. Sample Preparation and Analysis Using Gas Chromatography with Flame Ionization Detection

Upon sampling, fresh plant material was placed in a Fisher IsoTemp (Thermo Fisher Scientific Inc., Waltham, MA, USA) oven set to 90 °C overnight, resulting in a dry sample that was operationally defined as being easily crumbled between two fingers. The dried plant material was passed through a #10 sieve to comminute and to remove seeds (if any) and stems. The comminuted samples were mixed manually, and then two portions of about 200 mg each were massed and extracted with about 25 g of methanol, which was also massed. The average of the two test results was used if the relative percent difference between the two was <15%. If not, extracts were reinjected, and/or the sample was reprepared.

The comminuted samples were extracted in methanol with vigorous shaking and then analyzed directly by using an Agilent 7890A (Agilent Technologies Inc., Santa Clara, CA, USA) gas chromatograph equipped with a flame ionization detector (GC-FID), Rxi-35sil MS column, 15 × 0.25 × 0.25 m (Restek part #13820) and inlet liner (split type, cup design with wool packing, Supelco (Sigma-Aldrich, St. Louis, MO, USA [part #2048201]). The FID was operated at 350 °C with hydrogen at 40 mL/min, air at 400 mL/min and makeup flow at 45 mL/min. A 5 µL injection was made into an inlet at 250 °C by using a split injection (50:1) with a split flow of 100 mL/min. The initial oven temp was 225 °C, held for 0.1 min, ramped at 10 °C/min to 275 °C, held for 0 min, ramped to 325 °C at 25 °C and held for 2.9 min. The total run time was 10 min, with an elution order of CBD (~3.0 min), THC (~3.6 min) and CBN (~4.1 min). The THC and CBD content were analyzed directly as a single value that included the THCA and CBDA. The conversion of CBDA and THCA to the neutral forms upon hot injection was found to be sufficient, at least for extracts that contained a matrix, by the analysis of the certified reference material.

The calibration standards of THC and CBD were prepared gravimetrically in methanol by using an initial stock standard containing THC, CBD and CBN. The top calibrator was the initial stock standard (about 1263 µg/g), and the bottom calibrator was ~10 µg/g for THC and CBD with exact concentrations dependent upon the specific preparation. The instrument calibration is verified every 6 months, recalibrated if necessary and verified daily by using the independent calibration standard of THC only prepared at ~50 µg/g. CBN is generally regarded as a breakdown product of THC, which may indicate an aged product, and while part of the standard, it was not tracked.

The testing was shown to be in accordance with the controls set forth by the laboratory’s ISO/IEC 17025:2017 accredited quality-management system. The method-validation data showed that the measurement uncertainty associated with extraction and instrumentation was 7.4% for THC and 4.1% for CBD (assessed as the %RSD times a coverage factor of 2). These values meet the standard method performance characteristics listed in AOAC SMPR 2019.003 [40]. The method was assessed by the laboratory’s accreditation body in January of 2021 and is now included in the CAES Department of Analytical Chemistry scope of accreditation. Participation in annual external proficiency testing through the University of Kentucky has demonstrated that test results are appropriate for the determination of total delta-9 THC and total CBD on a continuing basis.

### 4.5. Data Analysis

Excel was used for basic data analysis and graphing while SigmaPlot 14 was used for conducting the analysis of variance. In SigmaPlot (Version 14.0), the one-way repeated-measures analysis of variance was used to compare the THC and CBD content of the bud material. SigmaPlot (Version 14.0) is preferred as it performs model adequacy checking and calculates the power of the test as well as generates the 95% confidence interval based on the standard error for the ratio of CBD to THC.

## 5. Conclusions

We demonstrate that hemp cultivars have a high probability of exceeding the legally allowed level of THC at full maturity. Additionally, we show that THC and CBD levels rise rapidly over a one-to-two-week time frame in the early fall for New England, which will make timing the harvest, versus relying on seed genetics, difficult. We demonstrated the occurrence of significant plant-to-plant variability in the levels of THC within the same genotype or cultivar. Therefore, to better represent small lots of hemp, sampling agents may need to collect more than the minimum number of plants required per the AMS guideline. The final rule specifies that the range of the test result is calculated based on the measurement uncertainty and that a field lot passes if the lower range of the test result is <0.3% total Delta-9 THC. However, the final rule does not provide guidance on what sources of uncertainty are included in the calculation, and it is probably difficult for laboratories to estimate uncertainty due to field sampling, which this study shows may be significant based on the observed within-cultivar plant-to-plant viability. Given the interlaboratory comparison data and plant heterogeneity and field sampling issues, the measurement uncertainty applied to THC test results for compliance levels is probably in the 30% to 50% range. Laboratories and their regulatory partners should discuss how measurement uncertainty is applied to test results for compliance purposes. Importantly, consumers should be aware that the ratio of CBD to THC in hemp cultivars is approximately 20:1 and that ratio is likely maintained in the end products. Additionally, hemp may be sold as individual buds, which may lead to unintended psychoactive effects. Although a previous study [11] indicated that THC and CBD production is almost entirely controlled by genetics, our data highlight the inherent difficulty in defining a hemp crop based on a test result versus the plant genetics, especially given the uncertainty around the collection of the sample, laboratory variability and plant-to-plant maturity.

## Figures and Tables

**Table 1:** The total delta-9 THC (%) for every plant within the cultivar demonstrates a high level of variability between individual plants on a given day.

Cultivar	YS17	SP7	CA20	EE	CW	BX
Average	0.962%	0.863%	0.702%	0.411%	0.484%	0.339%
STDEV	1.97	1.35	0.077	0.054	0.104	0.039
Min	0.135	0.361	0.544	0.240	0.116	0.248
Max	7.48	8.38	0.836	0.483	0.685	0.447
%RSD	205%	157%	11%	13%	22%	11%

**Table 2:** The total delta-9 CBD (%) for every plant within the cultivar demonstrates a high level of variability between individual plants on a given day.

Cultivar	YS17	SP7	CA20	EE	CW	BX
Average	4.66%	16.3%	18.5%	10.5%	13.3%	8.70%
STDEV	2.80	2.64	2.12	1.47	2.66	1.00
Min	0.794	8.28	14.1	5.97	2.68	6.04
Max	12.3	21.3	22.8	12.7	17.5	11.43
%RSD	60%	16%	11%	14%	20%	12%

**Table 3:** Hemp cultivars planted at the experimental farm.

Identifier	Cultivar	Supplier	Indoor Planting Date	Outdoor Planting Date	No. Plants
AEB	Abacus Early Bird	Hemp Logic	25 May 2020	25 June 2020	35
CAB	Cherry Abacus	Hemp Logic	25 May 2020	25 June 2020	27
AB20	Cherry Abacus 2.0	Hemp Logic	25 May 2020	25 June 2020	32
AE20	Abacus Early Bird 2.0	Hemp Logic	25 May 2020	25 June 2020	30
CA20	Cherry Abacus 2.0	Hemp Logic	25 May 2020	25 June 2020	31
SP7	Spec 7	Hemp Logic	25 May 2020	25 June 2020	33
YS17	Youngsim10 17	CO Agricultural Seed Solutions	25 May 2020	25 June 2020	14
YSWF	Youngims10-WF	CO Agricultural Seed Solutions	25 May 2020	25 June 2020	33
EE	Eighty Eight	Northwest Cultivation	3 May 2021	9 June 2021	25
CW	Cherry Wine	Fortuna Hemp	3 May 2021	9 June 2021	46
BG	Bhutan Glory	Northwest Cultivation	3 May 2021	9 June 2021	8
MR	Merlot	Fortuna Hemp	23 May 2022	27 June 2022	34
BX	BAOX	Fortuna Hemp	23 May 2022	27 June 2022	30
WF	Wife	Fortuna Hemp	23 May 2022	27 June 2022	31

## Data Availability

The datasets generated and/or analyzed during the current study are available from the corresponding author upon reasonable request.
